# Leukoencephalopathy and cerebral edema as the presenting manifestations of SLE in an ANA-negative adolescent female: a case report and review of literature

**DOI:** 10.1186/s12969-020-00449-2

**Published:** 2020-07-13

**Authors:** Alexandra Theisen, Paroma Bose, Christina Knight, Melissa Oliver

**Affiliations:** Pediatric Rheumatology, Riley Hospital for Children, 699 Riley Hospital Drive, Suite 307, Indianapolis, Indiana 46202 USA

**Keywords:** Neuropsychiatric systemic lupus erythematosus, Cerebral edema, Leukoencephalopathy, Prozone effect, ANA

## Abstract

**Background:**

Systemic lupus erythematosus (SLE) is an autoimmune disease with various clinical manifestations involving multiple organ systems. Neuropsychiatric manifestations of SLE have been associated with increased morbidity and mortality, thus it is important to recognize and diagnose the disease entity and treat early. When neuropsychiatric symptoms are involved, typically there are many other systemic features to aid in the diagnosis of SLE. Many autoantibodies have been discovered and are used to help diagnose SLE. The antibody present in most cases of pediatric SLE, as well as in many other rheumatic diseases, is the nonspecific antinuclear antibody (ANA). The ANA is a commonly used screening tool by primary care physicians when evaluating a patient with a possible rheumatic disorder. However, a small subset of SLE patients, 1–5%, present with a negative ANA, and it is important to keep SLE on the differential diagnosis in specific instances when a thorough infectious, metabolic and neurological workup has been completed and proven to be inconclusive.

**Case presentation:**

This case involves a Hispanic adolescent female with a negative ANA who presented with diffuse cerebral edema secondary to leukoencephalopathy due to SLE with central nervous system involvement. She was normotensive on presentation and relatively symptom free aside from headache. She had an extensive workup while inpatient involving metabolic, infectious disease, rheumatology, and neurology prior to obtaining the diagnosis of SLE. She was treated with cyclophosphamide and rituximab with appropriate disease response.

**Conclusions:**

A review of the literature revealed 12 cases with SLE presenting with or developing diffuse cerebral edema and/or leukoencephalopathy. Our patient’s case differs in that she was also ANA negative despite other autoantibody positivity. While she did have low complements and transient leukopenia, she did not present with other signs of organ involvement, which made the diagnosis of SLE with neuropsychiatric involvement quite challenging. We discuss the importance of keeping SLE on the differential diagnosis despite a negative ANA in complex cases after thorough workup has been unrevealing, and to consider initial screening with not only the ANA but also dsDNA and complements to avoid missed diagnoses.

## Background

Systemic lupus erythematosus (SLE) is a chronic inflammatory autoimmune disease characterized by multisystem clinical manifestations and associated autoantibodies, most commonly an antinuclear antibody (ANA) which is present in up to 95–99% of cases of pediatric SLE. Neuropsychiatric involvement in SLE (NPSLE) includes both the central and peripheral nervous system manifestations such as stroke, seizures, myelopathy, chorea, and psychosis, and more subtle findings such as mood disorders, cognitive impairment, and headaches [1–5]. Currently, there are 19 NPSLE syndromes as defined by the American College of Rheumatology [4, 6]. The prevalence of neuropsychiatric manifestations in various cohorts ranges from as low as 20% to as high as 95% [1, 3, 4]. In 25% of pediatric patients with SLE-related CNS disease, the initial symptom will be at presentation, and approximately 70% of these children will have CNS manifestations within the first year of diagnosis of SLE [7]. Neuropsychiatric lupus (NPSLE) has been associated with increased morbidity and mortality, thus is it extremely important to recognize and treat early if present. The most frequent NPSLE manifestations are headaches, psychiatric manifestations (including mood disorders, psychosis, cognitive dysfunction, and acute confusional state), cerebrovascular disease and seizures [4–7].

We present a case of pediatric SLE with primarily neuropsychiatric symptoms manifesting as cerebral edema secondary to acute leukoencephalopathy. Cerebral edema and leukoencephalopathy are rare reports as manifestations of NPSLE in the literature. Many of these patients already carried a diagnosis of SLE, previously had manifestations of NPSLE, and/or had other systemic symptoms related to their disease (Table 1). Furthermore, many of these patients were ANA-positive, which made this case especially challenging as our patient was ANA negative.

**Table 1:** Historical SLE cases in the literature with diffuse leukoencephalopathy

Patient (ref)	Age/Sex at presentation	Initial Presentation	Previous diagnosis of SLE	Previous neurological involvement	Neuroimaging	Positive ANA?	Other Ab Results Reported	Treatment	Outcome
1 [8]	38yo/F	Severe headache, syncope	YES	NO	CT: diffuse cerebral edema MRI: diffuse white matter hyperintensities	YES (1:2560)	-anti-dsDNA	3 day pulse-dose steroids→ oral prednisone, plaquenil	Herniation → death
2 [9]	11yo/F	Malar rash, photosensitivity, prolonged fever, hemolysis, generalized convulsions, unconsciousness	NO	N/A	MRI: high signal intensity in b/l basal ganglia and thalami, hyperintensities in deep white matter, pons, b/l caudate heads, putamens, thalami	YES	+anti-dsDNA +anti-ssDNA +anti-RNP +anti-Smith +anti-SSA	3 day pulse-dose steroids, IV 500 mg/day methylprednisolone	Return to baseline 1 year after insult
3 [8, 10]	14yo/F	HA 1 mo, progressive vomiting 1 week, abducens palsy 5 days	YES	NO	CT: Diffuse white matter hypodensity without ventricular dilatation. MRI: diffuse white matter hyperintensities	YES (1:320)	Unknown	3 day pulse-dose steroids w/steroid taper, ranitidine, plaquenil 200 mg.	No further recurrence, stable neurologically
4 [11]	35yo/F	Headache, mild Papilledema, skin eruption, fever	NO	N/A	MRI: diffuse hyperintense white matter lesions	YES	+anti-dsDNA	Unknown	Unknown
5 [12]	49yo/F	5wk constant HA, AMS, somnolence	YES	YES	CT: diffuse cerebral edema, small SAH MRI: diffuse sulcal hyperintensity	YES	+anti-dsDNA	Mannitol, 7 day high-dose steroids, IVIG, steroid taper	4 weeks from discharge, no recurrence
6 [13]	28yo/F	fever, malaise, facial edema, diplopia	NO	N/A	MRI: asymmetrical, multifocal high signal intensity lesions in subcortical white matter Gadnolinium: leptomeningial enhancement	Unknown	Unknown	3 days high-dose steroid pulse	Unclear
7 [14]	7yo/F	4 days ataxia, diplopia, morning vomiting; 1 yr hx of HA, recurrent vomiting, cognitive dysfunction	NO	N/A	CT: bilateral widening of the horizontal sulcus of cerebellum MRI: multiple cortico-subcortical lesions in both cerebral hemispheres with increased signal intensity.	YES (1:5120)	+anti-dsDNA -anti-RNP -anti-Smith -anti-Ro -anti-La -anti-mitochondrial	steroid pulse monthly, Cyclophosphamide monthly, continuous oral prednisolone	stabilization w/residual ataxia, dysmetria, psychomotor slowing.
8 [15]	32 yo/F	Nausea, vomiting, diplopia	NO	N/A	CT: diffuse cerebral edemaMRI: bilateral symmetric diffuse FLAIR hyperintensities Cerebral angiogram: no vasculitis	YES (1:1280)	+anti-dsDNA +anti-Smith	IV steroid pulse, Plasmapheresis, Cyclophosphamide, Acetazolamide, Mannitol, Hypertonic saline, hypothermia	Recalcitrant cerebral edema, sepsis, multi-organ failure ➔ death
9 [15]	29 yo/F	Loss of consciousness	YES	Unknown	CT: diffuse cerebral edema Cerebral angiogram: negative for vasculitis	Unknown	Unknown	Hydroxychloroquine, mycophenolate, IV methylprednisolone, IVIG	Recalcitrant cerebral edema ➔ death by neurologic criteria
10 [8, 16]	56 yo/F	Generalized macular rash, raynaud’s phenomenon, diarrhea, steady neurologic decline, dysphagia, pleural effusions, lymphopenia	NO	N/A	CT: normal MRI: extensive, confluent hyperintensity of the cerebral and cerebellar white matter	NO	+anti-dsDNA -anti-ENA -anti-Smith -anti-RNP -anti-La -anti-Ro	80 mg oral prednisone daily	Improvement of speech, swallowing. 1 year later ➔ mild hypophonia, some memory trouble
11 [8, 17]	35 yo/F	erythematous rash, polyarthropathy, Headache, photophobia,memory impairment	NO	N/A	CT: diffuse, uniform low attenuation in the white matter.	YES (1:320)	+anti-dsDNA	Oral prednisone, azathioprine	Improvement with oral prednisone
12 [8, 18]	41 yo/M	HA, vertigo, proteinuria, anemia, papilledema, retinal bleeding	YES	Unknown	Brain CT: diffuse brain edema MRI: diffuse white matter hyperintensities	YES	+anti-Smith	IV methylprednisolone, osmotic diuretics	Improvement in symptoms

## Case presentation

A 13-year old previously healthy Hispanic female presented with 1 week of nighttime fevers (Tmax 38.8 degrees Celsius orally), 3–4 days of occipital headache with blurry vision, and 1 day of neck pain. Initial vital signs were normal, including her blood pressure. She was afebrile and awake, alert, and oriented. Her physical exam was significant for grade IV papilledema on ophthalmologic exam (of note, papilledema is graded on a scale of I-V, V being most severe; Grade IV is characterized by loss of major vessels on the optic disc on examination). She otherwise had a normal neurologic exam, without meningeal signs. All other systems were normal. Her initial differential was most concerning for infectious, metabolic or neurologic causes. Initial laboratory studies showed leukopenia with a white blood cell count of 3.9 k/cubic mm (normal value 5–10 k/cubic mm), normocytic, normochromic anemia (Hgb 10.4), and normal platelet count. Leukopenia was noted on 2 occasions but was not sustained and resolved prior to treatment. Her erythrocyte sedimentation rate (ESR) and C-Reactive protein (CRP) were normal (see Table 2). Her urinalysis was without proteinuria or hematuria. She had normal renal function, electrolytes, toxicology screening, thyroid studies, folate, and vitamin B12 levels. Initial head CT showed diffuse cerebral swelling without herniation. Follow up brain MRI/MRA with and without contrast showed “symmetric diffuse T2 hyperintensity on the white matter of both cerebral hemispheres, brainstem, corpus callosum and cerebellar white matter with mild cerebellar tonsillar ectopia and no mass or midline shift”. This was consistent with diffuse brain swelling secondary to acute leukoencephalopathy. Brain MRA without contrast was without abnormalities. Due to severity of her cerebral edema, a lumbar puncture was not pursued. Despite her imaging, she was very well appearing and only complaining of occasional headache.

**Table 2:** Rheumatologic lab results and normal values

Lab Test	Patient Value	Normal Value
CRP	< 0.5 mg/dL	< 1.0 mg/dL
ESR	9 mm/hr	0–20 mm/hr
Complements (C3, C4)	C3: 12 mg/dL C4: 2 mg/dL	C3: 65–180 mg/dL C4: 13–52 mg/dL
beta 2-glycoprotein IgA QN	7.0 Units/mL	0.0–6.9 Units/mL
beta-2 glycoprotein IgG QN	5.8 Units/mL	0.0–6.9 Units/mL
beta-2 glycoprotein IgM QN	1.1 Units/mL	0.0–6.9 Units/mL
anti-cardiolipin antibody IgA	5.9 APL Units	> 22 APL Units may be clinically significant
anti-cardiolipin antibody IgG	6.5 GPL Units	0.0–9.9 GPL Units
anti-cardiolipin antibody IgM	4.8 MPL Units	0.0–9.9 MPL Units
lupus anticoagulant	31.6 s	24.3–42.6 s
ANA	< 1:40	< 1:80
Anti-dsDNA (on presentation)	82.2 IU/mL	0.0–9.9 IU/mL
Anti-dsDNA (13 months after treatment)	8.6 IU/mL	0.0–9.9 IU/mL
Anti-Smith	> 8.0	> 1.0 Test Value Positive
Anti-SSA EIA	> 8.0	> 1.0 Test Value Positive
Anti-SSB EIA	1.2	> 1.0 Test Value Positive
Anti-RNP	2.1	> 1.0 Test Value Positive
Anti-Smith/RNP EIA	5.7	> 1.0 Test Value Positive
Anti-Ribosomal P EIA	> 8.0	> 1.0 Test Value Positive
Anti-Neuronal serum Ab	> 400 Units	0–54 Units

During her hospitalization, multiple subspecialty services were involved in her care including neurosurgery, neurology, genetics, metabolism, infectious disease, ophthalmology, and rheumatology. Infectious studies were significant for positive Ebstein-Barr Virus (EBV) Nuclear Antigen IgG, Early Antigen IgG, Viral Capsid Antigen IgG and negative Viral Capsid Antigen IgM, positive Cytomegalovirus (CMV) IgG and IgM, and positive Mycoplasma IgG and IgM. Immunofluorescent antibody testing for mycoplasma pneumoniae IgM was negative. Arbovirus panel testing (which includes STL Encephalitis virus, California encephalitis virus, eastern equine virus, western equine virus) was sent twice and was inconclusive on both occasions due to specimen producing a “non-specific fluorescence”. Serum CMV PCR, EBV PCR, herpes simplex (HSV) virus type 1 and 2, cryptococcus blood antigen, human Immunodeficiency virus (HIV) 1 and 2 antibody and antigen, influenza A/B, adenovirus, parainfluenza 1–4, chlamydia pneumoniae, bocavirus, coronavirus, respiratory syncytial virus (RSV) A/B, rhinovirus/enterovirus, metapneumovirus, Lyme disease serologies, Brucella IgM/IgG, and West Nile IgM/IgG were all negative/unremarkable. TB spot testing (blood test based on IFN-gamma responses to Mycobacterium tuberculosis-specific antigens) was negative. An extensive metabolic workup was completed and results were normal, including serum amino acids, urine organic acids, lactate, pyruvate, very long chain fatty acids, lysosomal panel, carnitine, homocysteine, and CK. An MR spectroscopy was completed which showed “no significant abnormal elevated lactate peaks in the white matter basal ganglia”, which does not support a metabolic disorder. The MR spectroscopy did comment on “elevation of the glutamine/glutamate complex in short echo MRS in both the left frontal lobe and right basal ganglia” which can be seen with urea cycle disorders but typically in the more subacute/acute phase. Upon discussion with our metabolic team, her lab workup and imaging did not support a urea cycle disorder or other metabolic disorder. Neurology workup was limited due to severity of her cerebral edema and inability to obtain CSF studies.

She was managed medically for her cerebral edema with sodium supplementation for therapeutic hypernatremia and acetazolamide. She remained afebrile until day 4 of her hospitalization, however on hospital days 5, 6, and 7 she developed fevers. At this time, our Infectious Disease team initiated doxycycline for presumed mycoplasma infection due to positive IgG and IgM mycoplasma titers.

Throughout her hospitalization, she remained relatively normotensive (SBP/DBP: 91–122/52–83). She did have occasional blood pressure readings that were above her 95%ile but less than SBP 130. However, these readings were never persistent and did not require any medication. On day 7 of her hospital stay, she had acute deterioration and became unresponsive and obtunded, requiring emergent intubation. Her blood pressure at this time was slightly elevated at 142/95. A repeat head CT done at that time showed stable cerebral edema without new changes or herniation. EEG was negative for seizure activity. She was extubated to room air within 24 h and remained stable throughout the remainder of her hospitalization. Her blood pressure on day 8 of hospitalization was 135/86–139/86, after which she returned to being normotensive.

Rheumatology was consulted on day 8 of her admission for concern for CNS vasculitis or other autoimmune etiologies. She had an initial rheumatologic workup (see Table 2) that included ANA of < 1:40 by immunofluorescence (IF), but elevated anti-dsDNA (82.2), low C3 [19] and low C4 [2]. Antiphospholipid antibodies were significant for beta 2-glycoprotein IgA 7.0 (reference range being < 7.0), with beta-2 glycoprotein IgG and IgM negative, anti-cardiolipin antibodies negative, and lupus anticoagulant negative. The hypocomplementemia and positive anti-dsDNA antibody prompted further evaluation, specifically for SLE. Additional lab testing revealed positive Smith (> 8), SSA (> 8), SSB (1.2, reference range < 1.0), RNP (2.1, reference range < 1.0), Ribosomal P (> 8) and neuronal (> 400, reference range 0–54) antibodies. ANA was repeated and again noted to be < 1:40 by IF (Reference Table 2 for rheumatologic workup). She was ultimately diagnosed with SLE with CNS involvement. At this point in her disease process she had no other apparent organ involvement or other SLE features.

**Fig. 1:**
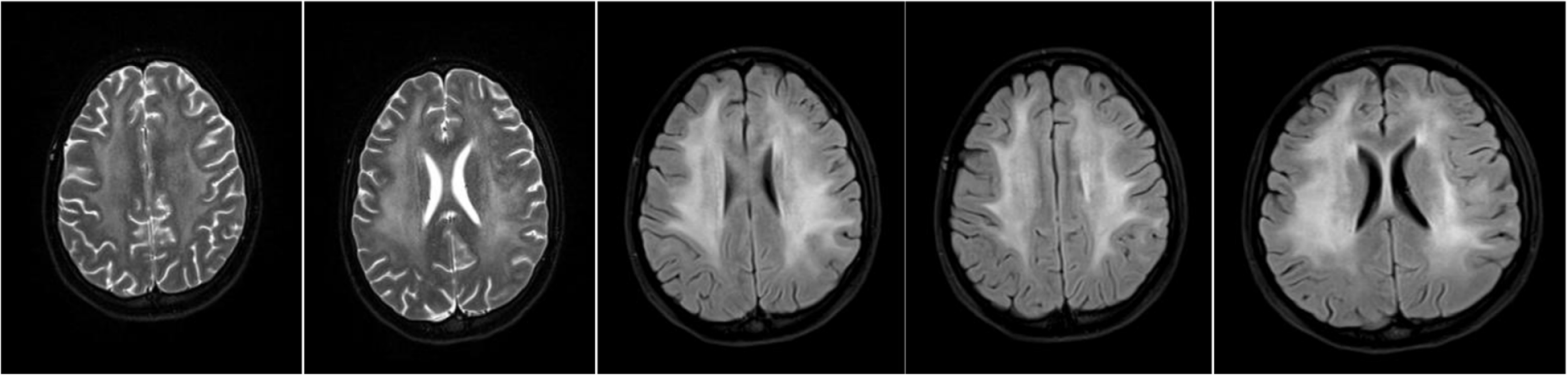
MRI brain with/without contrast

She initiated treatment for SLE and received 5 days of IV methylprednisolone (1 g) in addition to IV cyclophosphamide (initial dose 500 mg/m2) and IV Rituximab (500 mg/m2). Repeat MRI prior to discharge showed stable cerebral edema and leukoencephalopathy (Fig. 1). Her headaches subjectively improved with therapy, and repeat ophthalmologic exam showed improvement of papilledema from grade IV to grade II-III by time of discharge. She was discharged home on day 19 of hospitalization on prednisone 60 mg daily, hydroxychloroquine, and acetazolamide, with the outpatient management plan of second Rituximab infusion and monthly cyclophosphamide infusions and with IV solumedrol pulse dose therapy.

Figure 1 formal read: “Symmetric and confluent T2 intensity within white matter. Involvement of the corpus callosum splenium, internal and external capsule, brainstem at the dorsal tegmental tracts of the pons. Moderate degree of gray matter involvement at the anterior temporal lobe on the right and posterior left frontal lobe around the area of the precentral gyrus. No abnormal areas of reduced diffusion”. This was our patient’s resultant MRI after completing treatment prior to discharge.

## Discussion

This case report describes a pediatric patient with a new diagnosis of ANA-negative SLE with the initial findings of diffuse cerebral edema and acute leukoencephalopathy on imaging, characterized symptomatically only by headache, blurry vision and Grade IV papilledema on examination. Her fevers were thought to be due to active lupus as opposed to a CNS manifestation, largely because her cerebral involvement was quite diffuse as opposed to focused near the hypothalamus, where temperature regulation takes place. Hypothetically, if the fevers were secondary to CNS involvement she would have spiked fevers much more frequently given the severity of her CNS involvement on imaging. Instead, she spiked fevers intermittently. According to the literature, the finding of diffuse cerebral edema with or without leukoencephalopathy in NPSLE is extremely rare, and if present, develops later in the disease course and typically with other systemic signs of the disease. SLE patients with concern for neuropsychiatric involvement most commonly present with headaches, seizures, stroke, depression, and/or cognitive dysfunction as the sign of central neurologic involvement [4–7]. There have been case reports of patients with isolated intracranial hypertension as the only sign of neuropsychiatric lupus, and a few with intracranial hypertension with associated leukoencephalopathy (see Table 1), but we were unable to find an instance of a patient with isolated diffuse leukoencephalopathy as the presenting sign of lupus upon initial diagnosis. Many other patients described had other systemic signs/symptoms and already carried the diagnosis of SLE. Furthermore, most cases in the literature had a positive ANA in their workup to further assist in the diagnosis of SLE. Additional risk factors associated with development or worsening of NPSLE include generalized SLE activity or damage, history of previous or concurrent other major NPSLE, and antiphospholipid antibodies [3, 4]. Our patient did not have any of these risk factors.

As shown in Table 1, most of the case reports reviewed described intracranial hypertension with or without leukoencephalopathy [8–18]. Of the three cases that were children (ages, 7y, 11y, and 14y), all had a positive ANA, and one out of the three pediatric cases had a previous diagnosis of SLE. All cases reported multiple other clinical manifestations of SLE in addition to CNS involvement, unlike our patient. Imaging findings reported were consistent in showing diffuse hyperintensities on MRI suggestive of leukoencephalopathy, similar to our patient. Patient outcome across case reports were variable, with some making a full recovery and others unfortunately succumbing to their disease. Various methods were used for treatment, with high-dose steroids being a unifying treatment choice.

The pathophysiology has been explored in SLE cases of idiopathic intracranial hypertension (IIH) with diffuse leukoencephalopathy. There are multiple theories, including the possibility of immune-complex mediated damage, autoantibodies interacting (either directly or indirectly) with antigens on neuronal cell membrane, intrathecal cytokine production, and microangiopathy [1, 2]. Various autoantibodies found in relation to increased incidence of NPSLE include anti-phospholipid antibodies, anti-ribosomal P antibodies, and microtubule-associated protein-2 antibodies. These autoantibodies theoretically target endothelial cells, prostacyclins, protein C-S complex, and platelets, leading to acute impact on coagulation and chronic proliferative vasculopathy [2]. Cranial MRI is currently the anatomic imaging modality of choice for these patients, and displays high sensitivity but low specificity for NPSLE. Most NPSLE patients (40–80%) show small punctate focal lesions in periventricular and subcortical white matter areas on imaging, not necessarily associated with diffuse brain edema as was the case with our patient, and cerebral angiography typically is normal [2]. A vast array of findings in the literature make it very difficult to determine exact pathophysiology of diffuse leukoencephalopathy with associated cerebral edema. Pathophysiology is likely multifactorial, involving autoantibody reactivity as well as an underlying propensity for cerebral damage.

Interestingly, our patient is also unique in that she was diagnosed with SLE but had a negative ANA test noted on two occasions during her hospitalization and again after discharge. Additionally, she had a positive dsDNA antibody, multiple antibodies to extractable nuclear antigens (ENA) and low complements (Table 2). When considering our patient’s infectious workup, this was possibly representative of a diffuse polyclonal B-cell response and cross-reactivity resulting in false positive mycoplasma, EBV, CMV and the abnormal fluorescent for California virus, western and eastern equine viruses, and St. Louis virus antibodies. Additional autoantibody testing was not pursued until a thorough infectious, metabolic and neurologic workup was negative or inconclusive. Upon discussion with our metabolic team, her lab workup and imaging did not support a primary metabolic disorder. Her MR spectroscopy did comment on “elevation of the glutamine/glutamate complex in short echo MRS in both the left frontal lobe and right basal ganglia” (which can be seen with urea cycle disorders but typically in the more subacute/acute phase). However, her glutamine, citrulline and arginine were normal on serum amino acids, and urine orotic acid was normal, which does not support a urea cycle disorder. L 2 hydroxyglutaric aciduria was noted to be highly unlikely in the context of normal urine organic acids and normal lysine on serum amino acids. Mitochondrial disorders were less likely due to normal lactate and normal alanine on serum amino acids. Possible hereditary leukodystrophies were considered, however without cognitive decline, motor deterioration, dysmorphism, hepatosplenomegaly, or abnormal tone this was considered less likely. APOPT1 gene (associated with cavitating leukoencephalopathy with cytochrome C oxidase deficiency) testing was sent and was negative/unremarkable. Whole exome sequencing did not identify any variants that could be interpreted to definitively explain her reported phenotype, decreasing the possibility of a hereditary leukoencephalopathy or underlying genetic defect.

Neurology followed her case closely. Due to significant cerebral swelling on imaging and tonsillar ectopia, they did not feel it was safe to do a lumbar puncture due to risk of herniation. Autoimmune encephalitis was a consideration in both neurology and rheumatology differential diagnosis. However, her neurologic exam remained normal, she was without seizure-like activity or behavioral changes, her EEG was without epileptiform discharges, and her imaging was notable for diffuse cerebral edema with leukoencephalopathy as opposed to imaging changes limited to the hippocampi (as seen in anti-α-amino-3-hydroxy-5-methyl-4-isoxazolepropionic acid (AMPA) antibodies) or limbic system (as seen in anti-voltage-gated potassium channel (VGKC) antibodies). She had no evidence of an underlying tumor or malignancy on imaging or lab testing, which can be associated with autoimmune encephalitis. During her hospital stay, an autoimmune encephalitis panel in serum was suggested, however her workup continued to return more convincing for SLE, so this testing was not pursued.

Ultimately, it was the evidence of hypocomplementemia and a highly positive dsDNA antibody that led to the additional autoantibody testing and ultimately her diagnosis, though it took an extremely thorough workup and evaluation by other subspecialties prior to reconsidering SLE as her diagnosis. The dsDNA testing in our laboratory is completed by immunofluorescent assay with Crithidia luciliae, a flagellate parasite containing circula dsDNA without other nuclear antigens in the kinetoplast [20]. Crithidia luciliae and Farr assay detect higher-avidity antibodies, with the greatest specificity for the diagnosis of SLE [20]. As our testing methods are quite specific, this prompted pursuing further testing for rheumatologic disease. She had multiple positive ENA antibodies (Smith, RNP, SSA, SSB), ribosomal P antibody and neuronal antibody on further evaluation (Table 2). Our patient did not meet classification criteria for SLE based on ACR, SLICC, or ACR/EULAR. However, her clinical picture, serologies and response to treatment support the diagnosis of SLE.

Our patient was treated with both Rituximab and Cyclophosphamide for her SLE. We chose to give Cyclophosphamide given the severity of her CNS involvement and inflammation with brain edema. Rituximab was also given because of our patients’ significant auto-antibody load. B cells have an important role in the pathogenesis of SLE and lead to significant autoantibody production. Rituximab effectively helps reduce this level of auto-antibodies. As the time to effectiveness for rituximab and CNS manifestations is unknown, it was important to treat very aggressively with both Cyclophosphamide and Rituximab. She responded well to these medications and was able to discharge home shortly after obtaining immunosuppressive therapy.

Our patient is a unique representation of SLE, but her case may suggest that for some specific instances, where thorough infectious, metabolic, and neurological testing are inconclusive, that ANA testing alone may not be sufficient for screening for SLE. There are few descriptions of such cases in the literature but some do describe the importance of screening patients with suspected rheumatologic disorders with more than just the basic ANA screen [21]. Testing with other markers specific to SLE disease activity, e.g. complement levels and anti-dsDNA, proved critical in the diagnosis of SLE for our patient. On treatment of her disease over time, her anti-dsDNA antibody decreased significantly in line with her disease activity (Table 2), confirming response to treatment.

Various studies propose several reasons as to why some patients with the diagnosis of SLE have negative ANA screenings. These include the prozone or hook effect, the entity of an ANA negative SLE patient, or technical issues with the ANA screen itself [19]. We believed the prozone effect may have been responsible for our patient’s negative ANA result. The prozone effect occurs in cases of very high antibody concentrations and is thought to be responsible for negative immunoassays that involve the detection of antigen-antibody complexes [22]. With these assays, there is dependence on agglutination to reveal the presence of the antibody and thus confirm a positive test. With the prozone effect, the antibody concentration is so high, it interferes with the clumping of antigen-antibody complexes resulting in a seemingly negative result. ANA testing was repeated in our patient on multiple occasions and was consistently reported as negative. The ANA test at our facility is conducted via immunofluorescence with HEp-2 cells as the substrate molecule. The American College of Rheumatology position statement on ANA testing states the use of IIF as the gold standard method for ANA screening, specifically IIF on HEp-2 cells [23]. Her ANA test being negative initially drew the diagnosis away from the possibility of SLE, and it ultimately took a very thorough negative workup in other subspecialties before the diagnosis of SLE was re-considered. If the prozone effect is the cause of our patient’s negative ANA result, future ANA tests for our patient could turn positive with treatment of the patient’s disease and reduction in antibody burden.

## Conclusion

Our patient presented with a very rare form of NPSLE, and in addition was found to be ANA-negative on serological testing making this a challenging diagnosis of SLE. Isolated diffuse cerebral edema and leukoencephalopathy in SLE has rarely been reported in the literature. Resolution is possible with appropriate therapy, but mortality is a major concern due to the diffuse and severe vasogenic edema with lymphocytic infiltration. Therefore, it is important to recognize diffuse cerebral edema with leukoencephalopathy as being on the differential for possible NPSLE manifestation, despite ANA results, in order to improve patient outcomes.

## Data Availability

Not applicable.

## Abbreviations

ANA: Antinuclear antibody
NPSLE: Neuropsychiatric systemic lupus erythematosus
SLE: Systemic lupus erythematosus
